# *Aspergillus niger* uses the peroxisomal CoA-dependent β-oxidative genes to degrade the hydroxycinnamic acids caffeic acid, ferulic acid, and *p*-coumaric acid

**DOI:** 10.1007/s00253-021-11311-0

**Published:** 2021-05-05

**Authors:** R. J. M. Lubbers, A. Dilokpimol, J. Visser, R. P. de Vries

**Affiliations:** Fungal Physiology, Westerdijk Fungal Biodiversity Institute & Fungal Molecular Physiology, Utrecht University, Utrecht, The Netherlands

**Keywords:** Beta-oxidation, Peroxisome, Hydroxycinnamic acids, Fatty acids, Aromatic compounds

## Abstract

**Abstract:**

Aromatic compounds are important molecules which are widely applied in many industries and are mainly produced from nonrenewable sources. Renewable sources such as plant biomass are interesting alternatives for the production of aromatic compounds. Ferulic acid and *p-*coumaric acid, a precursor for vanillin and *p*-vinyl phenol, respectively, can be released from plant biomass by the fungus *Aspergillus niger*. The degradation of hydroxycinnamic acids such as caffeic acid, ferulic acid, and *p*-coumaric acid has been observed in many fungi. In *A. niger*, multiple metabolic pathways were suggested for the degradation of hydroxycinnamic acids. However, no genes were identified for these hydroxycinnamic acid metabolic pathways. In this study, several pathway genes were identified using whole-genome transcriptomic data of *A. niger* grown on different hydroxycinnamic acids. The genes are involved in the CoA-dependent β-oxidative pathway in fungi. This pathway is well known for the degradation of fatty acids, but not for hydroxycinnamic acids. However, in plants, it has been shown that hydroxycinnamic acids are degraded through this pathway. We identified genes encoding hydroxycinnamate-CoA synthase (*hcsA*), multifunctional β-oxidation hydratase/dehydrogenase (*foxA*), 3-ketoacyl CoA thiolase (*katA*), and four thioesterases (*theA-D*) of *A. niger*, which were highly induced by all three tested hydroxycinnamic acids. Deletion mutants revealed that these genes were indeed involved in the degradation of several hydroxycinnamic acids. In addition, *foxA* and *theB* are also involved in the degradation of fatty acids. HcsA, FoxA, and KatA contained a peroxisomal targeting signal and are therefore predicted to be localized in peroxisomes.

**Key points:**

*• Metabolism of hydroxycinnamic acid was investigated in Aspergillus niger*

*• Using transcriptome data, multiple CoA-dependent β-oxidative genes were identified.*

*• Both foxA and theB are involved in hydroxycinnamate but also fatty acid metabolism.*

**Supplementary Information:**

The online version contains supplementary material available at 10.1007/s00253-021-11311-0.

## Introduction

Aromatic compounds are applied in many industries such as food and beverage, cosmetic, polymer, and pharmaceutical industries and are mainly produced from nonrenewable petroleum sources, which are slowly depleting. Therefore, alternative sources such as plant biomass are investigated for the production of aromatic compounds. For example, the hydroxycinnamic acid ferulic acid (4-hydroxy-3-methoxycinnamic acid) can be released from sugar beet pulp by feruloyl esterases from the industrial important fungus *Aspergillus niger* (Lesage-Meessen et al. 1999; Benoit et al. 2006). Ferulic acid is particularly interesting since it can be used as a precursor for vanillin which is an important aromatic compound for the food industry (Kaur and Chakraborty 2013; Banerjee and Chattopadhyay 2019). Vanillin can be produced from this compound using a two-step bioconversion process, in which *A. niger* converts ferulic acid to vanillic acid and *Pycnoporus cinnabarinus* converts vanillic acid to vanillin (Lesage-Meessen et al. 1996). More recently, it has been shown that ferulic acid released from pineapple leaves and crowns can be converted to vanillin and vanillic acid by *A. niger* (Tang and Hassan 2020). However, the metabolic pathway for this conversion remains unknown.

Aromatic metabolism in fungi has been studied for decades, but most studies are scattered over different fungal species (Milstein et al. 1983; Mäkelä et al. 2015; Lubbers et al. 2019c). Therefore, it remains largely unknown which pathways are present in a single species. The most complete overview of aromatic metabolic pathways is from *Aspergillus japonicus* (Milstein et al. 1983). However, not many genes involved in these pathways have been identified. Recently, several genes and enzymes of the aromatic metabolic pathways from *A. niger* have been identified and characterized (Lubbers et al. 2019a, b). It has been shown that the hydroxycinnamic acids, ferulic acid, *p-*coumaric acid (4-hydroxycinnamic acid), and caffeic acid (3,4-dihydroxycinnamic acid), were converted to vanillic acid, *p-*hydroxybenzoic acid, and protocatechuic acid, respectively (Lubbers et al. 2019b, 2020). However, it remains unknown which enzymes mediate the conversion of hydroxycinnamic acids to their benzoate forms.

In microorganisms and plants, multiple pathways have been described for the degradation of hydroxycinnamic acids, such as caffeic acid, ferulic acid, and *p-*coumaric acid (Supplemental Fig. S1) (Widhalm and Dudareva 2015; Lubbers et al. 2019c). The first pathway is the CoA-independent oxidative pathway, which is mostly observed in bacteria. Ferulic acid is decarboxylated to *p-*vinylguaiacol by a phenolic acid decarboxylase, after which it is reduced to vanillin (Mathew et al. 2007; Mishra et al. 2014; Furuya et al. 2015; Han et al. 2019). This pathway was observed in *Aspergillus luchuensis* (Maeda et al. 2018; Taira et al. 2018). The second pathway is the CoA-dependent non-oxidative pathway, which is well studied in bacteria (Priefert et al. 1999; Plaggenborg et al. 2003; Yang et al. 2013; Fleige et al. 2016). Ferulic acid is converted to feruloyl-SCoA by a feruloyl-CoA synthetase (also known as a very long-chains acyl-CoA synthetase). A multifunctional enzyme called enoyl-CoA hydratase/aldolase (Ech) hydrates feruloyl-SCoA to 4-hydroxy-3-methoxyphenyl-β-hydroxypropionyl-SCoA (HMPHP-SCoA) and then hydrolyzes this compound to vanillin and acetyl-CoA. Finally, vanillin is converted to vanillic acid. This pathway has been suggested for the degradation of ferulic acid and *p*-coumaric acid in the fungus *P. cinnabarinus* (Falconnier et al. 1994; Alvarado et al. 2001). The third pathway is the CoA-dependent β-oxidative pathway which is well studied in plants and was also observed to occur in bacteria (Otani et al. 2014; Widhalm and Dudareva 2015). The first step in this pathway is similar to the CoA-dependent non-oxidative pathway. Feruloyl-SCoA is hydrated to HMPHP-SCoA followed by a dehydrogenase step to 4-hydroxy-3-methoxyphenyl-β-ketopropionic acid-SCoA (HMPKP-SCoA), which is catalyzed by a bifunctional hydratase/dehydrogenase enzyme. HMPKP-SCoA is then converted to vanillyl-SCoA by a 3-ketoacyl-CoA thiolase. Finally, vanillyl-SCoA is converted to vanillic acid catalyzed by a thioesterase. In *A. niger*, all three pathways have been suggested, but the genes involved have not been identified (Baqueiro-Peña et al. 2010; Srivastava et al. 2010; Lubbers et al. 2020). Interestingly, vanillin, an important intermediate for the CoA-dependent non-oxidative and CoA-independent oxidative pathway, has not been observed during the degradation of ferulic acid (Lesage-Meessen et al. 1996; Baqueiro-Peña et al. 2010; Lubbers et al. 2020). Therefore, we hypothesize that *A. niger* mainly uses the CoA-dependent β-oxidative pathway to degrade the hydroxycinnamic acids caffeic acid, ferulic acid, and *p*-coumaric acid. In this study, we aimed to identify the genes encoding the enzymes of the CoA-dependent β-oxidative pathway using whole-genome transcriptome data. Multiple genes were identified, and the encoded enzymes were shown to be involved in the degradation of the hydroxycinnamic acids. In addition, in silico localization prediction of the enzymes involved was performed.

## Materials and methods

### Strains, media, and culture conditions

All *A. niger* strains used in this study are shown in Table 1. To obtain conidia, the fungi were grown on complete medium (CM) (de Vries et al. 2004) agar plates at 30°C for 4 days. Spores were harvested with 10 mL *N*-(2-acetamido)-2-aminoethanesulfonic acid buffer, and 2 μL of suspension containing 10^3^ freshly isolated spores was inoculated (in duplo) on minimal medium (MM) (de Vries et al. 2004) agar (1.5% w/v) plates. Inoculated plates were incubated at 30°C for multiple days. MM plates for growth profile experiments were supplemented with aromatic compounds as sole carbon source. Due to the higher toxicity of ferulic acid and cinnamic acid, 2 mM was used for the growth profile, while 5 mM was used for the remaining aromatic compounds and fatty acids. All aromatic compounds, fatty acids and chemicals were purchased from Sigma Aldrich (Saint Louis, MO, USA).

**Table 1:** Strains used in this study**.** Numbers in brackets corresponds to the JGI *A. niger* NRRL3 protein ID number

Strain	CBS number	Genotype	Reference
N593 Δ*kusA*	CBS 138852	*cspA1*, *pyrG*, Δ*kusA*::*amdS*	Meyer et al. (2007)
Reference	CBS 145984	*cspA1*, *pyrG*, Δ*kusA*::*amdS*, Δ*pyrG*::*pyrG*	Lubbers et al. (2021)
Δ*hcsA* (5989)	CBS 146832	*cspA1*, *pyrG*, Δ*kusA*::*amdS*, Δ*hcsA*::*pyrG*	This study
Δ*katA* (5990)	CBS 146833	*cspA1*, *pyrG*, Δ*kusA*::*amdS*, Δ*katA*::*pyrG*	This study
Δ*foxA* (672)	CBS 146834	*cspA1*, *pyrG*, Δ*kusA*::*amdS*, Δ*foxA*::*pyrG*	This study
Δ*theA* (621)	CBS 146835	*cspA1*, *pyrG*, Δ*kusA*::*amdS*, Δ*theA*::*pyrG*	This study
Δ*theB* (1539)	CBS 146836	*cspA1*, *pyrG*, Δ*kusA*::*amdS*, Δ*theB*::*pyrG*	This study
Δ*theC* (2735)	CBS 146837	*cspA1*, *pyrG*, Δ*kusA*::*amdS*, Δ*theC*::*pyrG*	This study
Δ*theD* (6009)	CBS 146838	*cspA1*, *pyrG*, Δ*kusA*::*amdS*, Δ*theD*::*pyrG*	This study

### Transcriptome data

Transcriptome data was downloaded from NCBI gene expression omnibus. Data of *A. niger* N402 grown on caffeic acid, *p-*coumaric acid, *p*-hydroxybenzoic acid, and no carbon source was obtained from GEO accession number GSE134999 (Lubbers et al. 2019b). Data of *A. niger* N402 on ferulic acid was obtained from GSE135001 (Lubbers et al. 2020). The transcriptome data was analyzed as previously described (Lubbers et al. 2020).

### Deletion mutants

Gene deletion cassettes were constructed using fusion-PCR as described previously (Kowalczyk et al. 2017). The flanking regions contained 900–1000 bp upstream and downstream of the gene of interest, including an overlap of the selection marker orotidine-5′-phosphate decarboxylase (*pyrG*) that was amplified from *Aspergillus oryzae* RIB40 (Supplemental Table S1). The flanking regions and *pyrG* were combined by a fusion PCR as described previously (Lubbers et al. 2019b). *A. niger* N593 Δ*kusA* was transformed through protoplast-mediated transformation, and purification of the different transformants was performed as described previously (Kowalczyk et al. 2017). The gDNA from the deletion mutants was isolated, and the knockout was verified by PCR.

### In silico localization prediction

Amino acid sequences of HcsA, FoxA, KatA, TheA, TheB, TheC, and TheD were downloaded from JGI and uploaded to Deeploc (http://www.cbs.dtu.dk/services/DeepLoc/) and Loctree3 (https://rostlab.org/services/loctree3/) cellular localization prediction software (Goldberg et al. 2014; Almagro Armenteros et al. 2017). Deeploc software was used on the accurate setting, and Loctree3 was set for eukaryota.

### Phylogenetic analysis

To perform the phylogenetic analysis, the amino acid sequence of HcsA, FoxA, and KatA were used for BLASTP analysis on selected ascomycete and basidiomycete genomes (Supplemental Table S2). To reduce the amount of insignificant hits, a cutoff *E* = −40 was used. The amino acid sequences of 4-coumarate-CoA ligases (4CL) (Uniprot; 4CL1, Q42524; 4CL2, Q9S725; 4CL3, Q9S777) and 4-coumarate-CoA ligases-like (4CLL) (Uniprot; 4CLL1, Q9LQ12; 4CLL2, Q84P25; 4CLL3, Q3E6Y4; 4CLL5, Q84P21; 4CLL6, Q84P24; 4CLL7, Q9M0X9; 4CLL8, Q84P26; 4CLL9, Q84P23) from *Arabidopsis thaliana* and feruloyl-CoA synthetase (Uniport: fsc Q9RLD9) from *Pseudomonas* sp. were included as outgroup in the phylogenetic analysis of HcsA. As outgroup for FoxA, the peroxisomal fatty acid beta-oxidation multifunctional protein (Uniprot: AIM1, Q9ZPI6) from *A. thaliana* and 3-hydroxyacyl-CoA dehydrogenase (Uniprot: CHD1, J9YIZ4) from *Petunia hybrida* was included.

Amino acid sequences were aligned using the MUSCLE algorithm implemented in MEGA X with default settings (Kumar et al. 2018). Several amino acid sequences were curated manually or with gene prediction software, Augustus (Stanke et al. 2004) (Supplemental Table S3). The Maximum Likelihood trees were constructed using MEGA X with 500 bootstraps and were visualized with iTol (Kumar et al. 2018; Letunic and Bork 2019).

## Results

### Identification of the CoA-dependent β-oxidative pathway genes

To identify candidate genes involved in the CoA-dependent β-oxidative pathway, whole-genome transcriptome data from *A. niger* grown on caffeic acid, ferulic acid, *p-*coumaric acid, *p-*hydroxybenzoic acid, and protocatechuic acid were compared to the no carbon source control (Lubbers et al. 2019b, 2020).

The first step of the CoA-dependent oxidative pathway is catalyzed by feruloyl-CoA synthase or cinnamoyl-CoA ligase (very long-chain acyl-CoA synthase). In the *A. niger* genome, three genes (NRRL3_3742, NRRL3_4938, NRRL3_5989) are annotated as very long-chain acyl-CoA synthases. Only NRRL3_5989 was highly induced (FPKM ≥ 10, fold change (log2) ≥ 4, *p* ≤ 0.01) by caffeic acid, ferulic acid, and *p*-coumaric acid (Table 2). Therefore, NRRL3_5989 was selected as the candidate hydroxycinnamate-CoA synthase (*hcsA*). The second and third steps are catalyzed by a cinnamoyl-CoA hydratase/dehydrogenase or peroxisomal fatty acid β-oxidation multifunctional protein. In *Aspergillus nidulans*, a peroxisomal β-oxidation multifunctional enzyme (FoxA, AN7111) has been identified and is involved in the metabolism of very long-chain fatty acids (Maggio-Hall and Keller 2004). A BLAST search using the amino acid sequence of FoxA against the *A. niger* genome resulted in a single hit NRRL3_672 that showed the highest amino acid similarity (73.4%). NRRL3_672 is induced (FPKM ≥ 10, fold change (log2) ≥ 1, *p* ≤ 0.01) by caffeic acid, ferulic acid, and *p-*coumaric acid (Table 2). Therefore, NRRL3_672 was selected as a candidate for the β-oxidation hydratase/dehydrogenase (*foxA*). The fourth step of the pathway is catalyzed by a 3-ketoacyl CoA thiolase. Interestingly, the gene NRRL3_5990, neighboring the putative *hcsA* gene, is annotated as a 3-ketoacyl CoA thiolase and was also found to be highly induced under the same conditions (Table 2). Therefore, NRRL3_5990 was selected as candidate 3-ketoacyl CoA thiolase (*katA*). The fifth step of the CoA-dependent oxidative pathway is catalyzed by a thioesterase. Multiple genes are annotated as thioesterases, and four were induced by ferulic acid, *p*-coumaric acid, or caffeic acid. Therefore, we selected four genes (NRRL3_621, NRRL3_1539, NRRL3_2735, NRRL3_6009) to be further studied (Table 2) that were named *theA*, *theB*, *theC*, and *theD*, respectively.

**Table 2:** Transcriptome data from putative CoA-dependent β-oxidative pathway genes induced by aromatic compounds. Genes that were considered to be highly induced, based on the criteria of FPKM ≥ 10, fold change (log2) ≥ 4, *p* ≤ 0.01, are in bold. Induced genes following the criteria of FPKM ≥ 10, fold change (Log2) ≥ 1, *p* ≤ 0.01, are underlined. Fold change and *p*-values were calculated with DeSeq2 (Love et al. 2014)

NRRL3 gene ID	Ferulic acid			*p*-Coumaric acid			*p-*Hydroxybenzoic acid			Caffeic acid			Protocatechuic acid			No carbon source
	FPKM	FC (log2)	*p*-value	FPKM	FC (log2)	*p*-value	FPKM	FC (log2)	*p*-value	FPKM	FC (log2)	*p*-value	FPKM	FC (log2)	*p*-value	FPKM
5989 (*hcsA*)	**1800**	**6.83**	**0.00**	**1512**	**6.61**	**0.00**	85	2.45	0.00	**1687**	**6.78**	**0.00**	62	1.96	0.00	16
3742	87	-0.50	0.11	75	-0.69	0.02	110	-0.30	0.44	170	0.49	0.12	100	-0.49	0.18	149
4938	120	1.69	0.00	71	0.96	0.00	41	0.01	0.99	108	1.58	0.00	35	-0.24	0.45	44
672 (*foxA*)	2451	1.98	0.00	3526	2.51	0.00	563	-0.26	0.48	4246	2.79	0.00	348	-0.98	0.00	733
5990 (*katA*)	**8088**	**6.26**	**0.00**	**4345**	**5.42**	**0.00**	209	1.07	0.02	**4900**	**5.60**	**0.00**	157	0.63	0.25	105
621 (*theA*)	207	1.92	0.00	179	1.73	0.00	133	1.14	0.00	85	0.69	0.02	227	1.85	0.00	65
1539 (*theB)*	189	1.51	0.00	262	1.99	0.00	120	0.73	0.05	185	1.51	0.00	65	-0.18	0.75	78
2735 (*theC*)	210	2.90	0.00	147	2.41	0.00	37	0.27	0.98	117	2.10	0.00	24	-0.35	0.76	33
6009 (*theD*)	581	2.88	0.00	263	1.76	0.00	85	-0.01	0.48	212	1.47	0.00	97	0.13	0.39	93

*FC* fold change, *FPKM* fragments per kilobase million

### Deletion of putative CoA-dependent β-oxidative pathway genes results in reduced growth on hydroxycinnamic acids

**Fig. 1:**
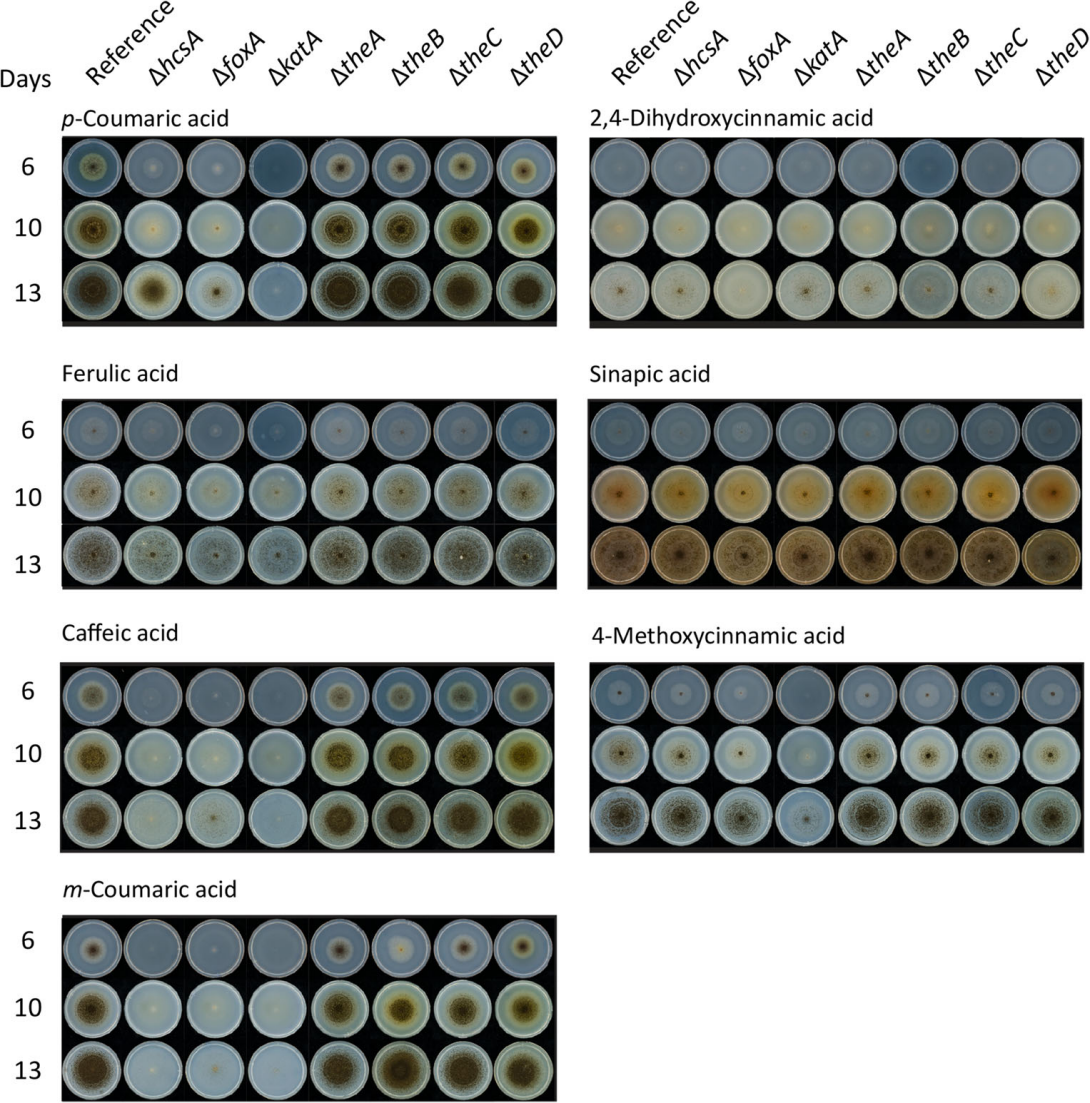
Phenotypic analysis of the deletion mutants on hydroxycinnamic acids after 6, 10, and 13 days of growth. MM agar plates with aromatic compounds as sole carbon sources were inoculated with spores and incubated at 30°C. The experiment was conducted in duplicate, and no difference between the replicates were observed

**Fig. 2:**
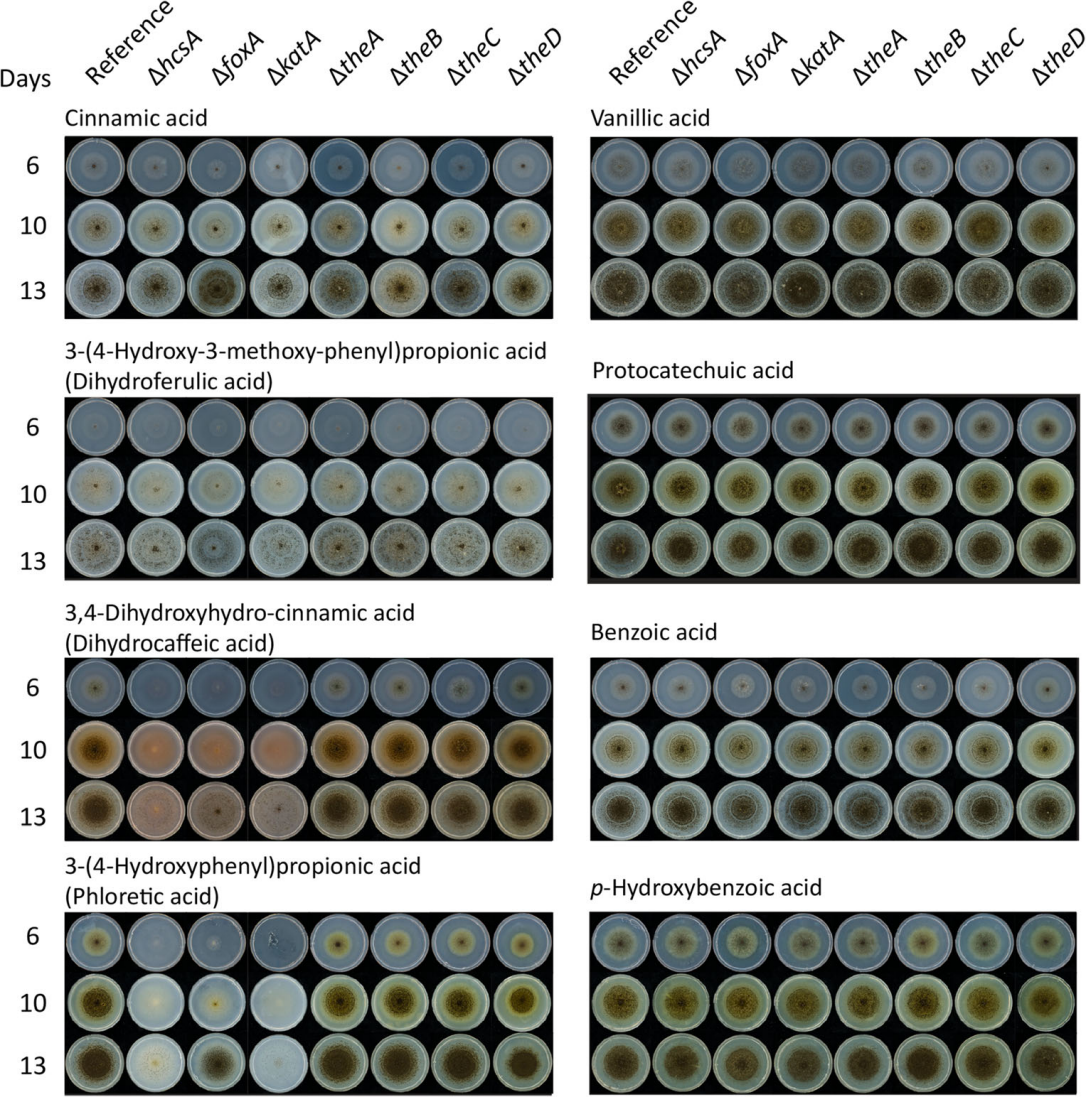
Phenotypic analysis of the deletion mutants on cinnamic acid, hydrocinnamic acids, and benzoic acids after 6, 10, and 13 days of growth. MM agar plates with aromatic compounds as sole carbon sources were inoculated with spores and incubated at 30°C. The experiment was conducted in duplicate, and no difference between the replicates were observed

To verify that the candidate genes encode the enzymes involved in the CoA-dependent β-oxidative pathway, deletion mutants were created and cultivated on several aromatic compounds as a sole carbon source. Deletion of *hcsA*, *foxA*, or *katA* resulted in reduced growth on the hydroxycinnamic acids: ferulic acid, *p*-coumaric acid, *m-*coumaric acid, caffeic acid, and the hydrocinnamic acids: 3-(4-hydroxy-3-methoxyphenyl)-propionic acid (dihydroferulic acid), 3,4-dihydroxyhydrocinnamic acid (dihydrocaffeic acid) (Fig. 1), and 3-(4-hydroxyphenyl)-propionic acid (phloretic acid) compared to the reference strain (Fig. 2). Interestingly, the growth is less reduced on ferulic acid and dihydroferulic acid compared to caffeic acid or *p-*coumaric acid. Deletion of *katA* resulted in more severely reduced growth on *p-*coumaric acid and 4-methoxycinnamic acid compared to *hcsA* and *foxA*. Deletion of *foxA* also resulted in reduced sporulation on 2,4-dihydroxycinnamic acid and reduced growth on 4-methoxycinnamic acid and cinnamic acid, but growth recovered overtime (Figs. 1 and 2). No phenotypes were observed on sinapic acid, benzoic acid, *p*-hydroxybenzoic acid, protocatechuic acid, or vanillic acid (Figs. 1 and 2).

The deletion of the thioesterases did not result in phenotypes on ferulic acid, caffeic acid, or *p-*coumaric acid. Only Δ*theB* showed a clear phenotype on *m-*coumaric acid, which became less severe after ten days (Fig. 1). The recovery and lack of phenotypes is probably due to the functional redundancy of thioesterases resulting in the compensation for the loss of *theB*.

### *foxA* and *theB* are also involved in the β-oxidation of fatty acids

In *A. nidulans*, FoxA has been reported to be involved in fatty acid metabolism (Maggio-Hall and Keller 2004). Therefore, a phenotypic screening was performed with the deletion mutants and the reference strain on fatty acids, i.e., eruric acid, oleic acid, hexanoic acid, and crotonic acid as sole carbon source.

**Fig. 3:**
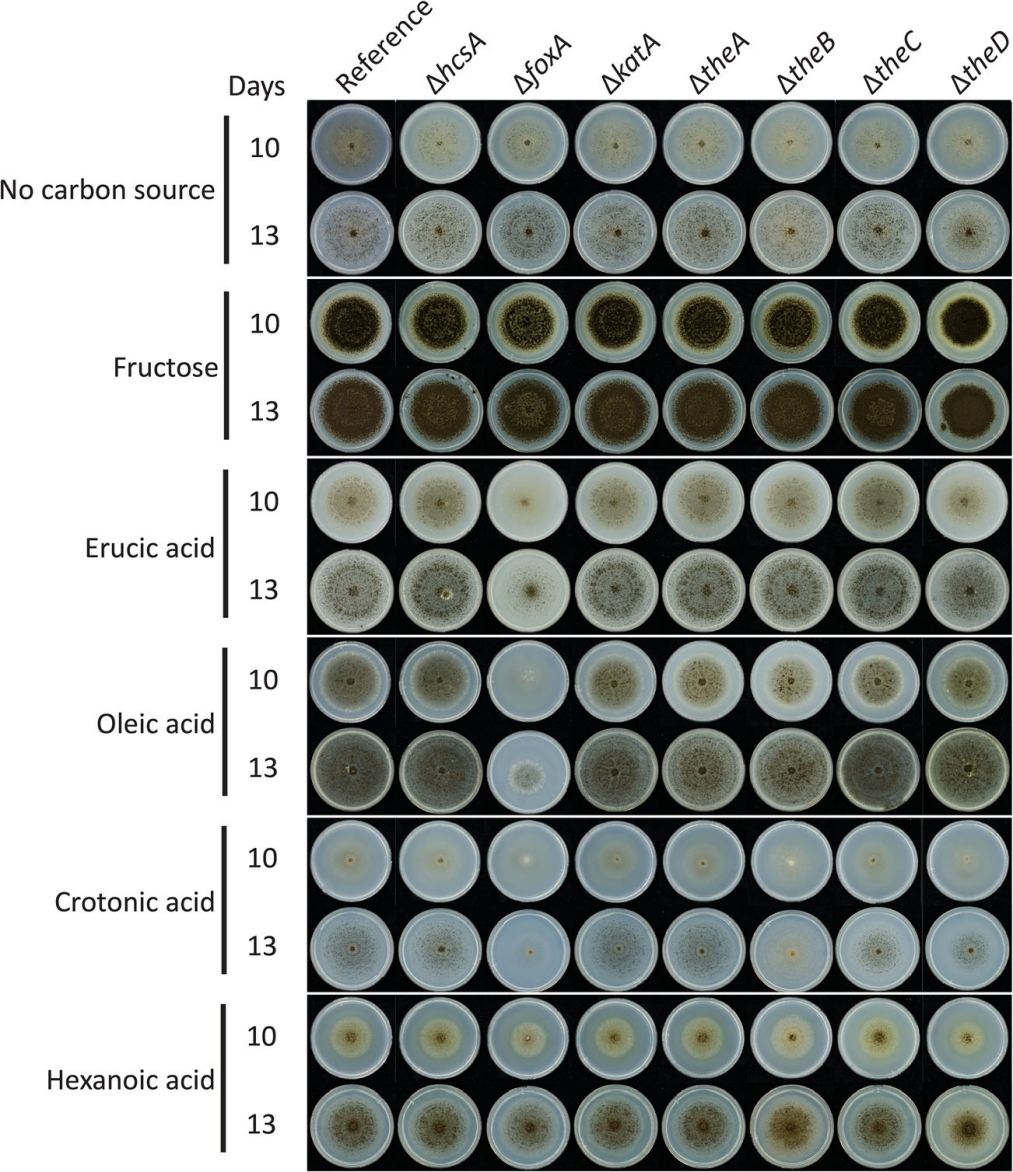
Phenotypic analysis of the deletion mutants on fatty acids after 10 and 13 days of growth. MM agar plates with fatty acids as sole carbon sources were inoculated with spores and incubated at 30°C. The experiment was conducted in duplicate, and no difference between the replicates were observed

All strains grew poorly on fatty acids, and no clear phenotypes were visible after 6 days of growth (data not shown). After 10 days, deletion of *foxA* resulted in reduced growth on eruric acid, oleic acid, and crotonic acid, but not on hexanoic acid (Fig. 3). Deletion of *theB* resulted in reduced sporulation on crotonic acid, while deletion of *theD* resulted in reduced sporulation on eruric acid. This indicates that FoxA and thioesterases are active on both aromatic compounds and fatty acids. Deletion of *hcsA*, *katA*, *theA*, or *theC* did not result in growth reduction suggesting that these genes are not involved in the fatty acid degradation.

### In silico localization of CoA-dependent β-oxidative pathway enzymes

The CoA-dependent β-oxidative pathway has been reported to be located in the peroxisomes (Widhalm and Dudareva 2015). Therefore, we used cellular localization prediction software to predict the localization of the CoA-dependent β-oxidative pathway enzymes (Table 3).

**Table 3:** In silico prediction of CoA-dependent β-oxidative pathway genes. The predictions were performed with Deeploc software (Almagro Armenteros et al. 2017) and LocTree3 (Goldberg et al. 2014)

		Deeploc		LocTree3	
Enzyme	Peroxisomal targeting signal	Predicted localization	Likelihood	Predicted localization	Expected accuracy (%)
HcsA	PTS1 (AKL)	Mitochondrial	0.728	ER membrane	84
FoxA	PTS1 (AKL)	Peroxisome	0.882	Peroxisome	88
KatA	PTS2 (RLTSIANQL)	Peroxisome	0.621	Peroxisome	89
TheA	-	Mitochondrial	0.578	Cytoplasm	88
TheB	-	Cytoplasm	0.605	Cytoplasm	84
TheC	PTS1 (SKL)	Cytoplasm	0.445	Peroxisome	84
TheD	-	Cytoplasm	0.661	Cytoplasm	86

The amino acid sequences of the CoA-dependent β-oxidative pathway enzymes were analyzed for the presence of a peroxisomal targeting signal (PTS) (Gould et al. 1989; Petriv et al. 2004). PTS1 was identified by the presence of the consensus [SAC][KRM][LM] in the C-terminus of the protein (Gould et al. 1989), while PTS2 was identified by using the consensus RXXXXX[HQ]L near the N-terminus of the protein (Petriv et al. 2004). FoxA and KatA contained a PTS and are predicted to be localized in the peroxisomes. The in silico prediction of HcsA was less certain, but it contains a PTS; hence, it is possible to localize in the peroxisomes. Only TheC was predicted to be localized in the peroxisome and contained a PTS, while TheA, TheB, and TheD are predicted to be localized in the cytoplasm or mitochondria.

### Peroxisomal genes are strongly induced by aromatic compounds

Based on the presence of PTS and the in silico prediction, both FoxA and KatA are possibly localized in the peroxisomes, which corresponds well with the observation that β-oxidative pathway occurs in the peroxisomes. To support this hypothesis, the transcriptome dataset was further analyzed for peroxisomal genes induced by ferulic acid, caffeic acid, and *p*-coumaric acid. Peroxisomal genes were selected and obtained from the JGI genome database based on gene annotations and GO terms.

Multiple peroxisomal genes were induced (FPKM ≥ 10, fold change (log2) ≥ 1, *p* ≤ 0.01) by ferulic acid, *p-*coumaric acid, and caffeic acid (Table 4). Two genes (NRRL3_7512 and NRRL3_9932) annotated as peroxisomal biogenesis factor 11 (Pex11) and one gene (NRRL3_6705) annotated as peroxisomal membrane protein 4 (Pex4) were strongly induced (FPKM ≥ 10, fold change (log2) ≥ 2, *p* ≤ 0.01) by ferulic acid, *p*-coumaric acid, and caffeic acid (Table 4). These results revealed that many peroxisomal genes are also induced by hydroxycinnamic acids and not only by long-chain fatty acids.

**Table 4:** Peroxisomal genes induced by ferulic acid, *p*-coumaric acid, or caffeic acid. Genes that were considered to be strongly induced when they followed the criteria of FPKM ≥ 10, fold change (log2) ≥ 2, *p* ≤ 0.01, are in bold. Induced genes followed the criteria of FPKM ≥ 10, fold change (log2) ≥ 1, *p* ≤ 0.01, and are underlined. Fold change and *p*-values were calculated with DeSeq2 (Love et al. 2014)

NRRL3 gene ID	Ferulic acid			*p-*Coumaric acid			Caffeic acid			NC	
	FPKM	Fold change	*p*-value	FPKM	Fold change	*p*-value	FPKM	Fold change	*p*-value	FPKM	Predicted JGI annotation
556	**486**	**2.02**	**0.000**	369	1.64	0.000	448	1.94	0.000	143	Mitochondrial/peroxisomal carrier protein
1504	62	1.25	0.000	52	1.00	0.000	45	0.83	0.001	31	Peroxisomal biogenesis factor 10 (Pex10)
2754	173	1.77	0.000	163	1.70	0.000	147	1.57	0.000	60	Peroxisomal membrane protein 7 (Pex7)
6116	245	1.60	0.000	196	1.30	0.000	195	1.31	0.000	97	Peroxisomal membrane protein 14 (Pex14)
6511	206	1.83	0.000	205	1.84	0.000	165	1.54	0.000	69	Peroxisomal biogenesis factor 3 (Pex3)
6705	**672**	**2.36**	**0.000**	**609**	**2.23**	**0.000**	**669**	**2.38**	**0.000**	154	Peroxisomal membrane protein 4 (Pex4)
6739	31	−0.69	0.128	85	0.70	0.122	176	1.72	0.000	62	Peroxisomal biogenesis protein 13 (Pex13)
6747	223	1.34	0.000	149	0.77	0.000	219	1.34	0.000	106	PTS 1 receptor family protein (Pex5)
6923	66	1.33	0.000	54	1.04	0.000	47	0.87	0.000	32	Peroxisomal biogenesis factor 1 (Pex1)
6994	516	1.43	0.000	370	0.97	0.000	362	0.96	0.000	230	Peroxisomal membrane protein 14 (Pex14)
7255	61	1.41	0.000	49	1.12	0.000	52	1.20	0.000	28	Peroxisomal biogenesis factor 6 (Pex6)
7512	**521**	**3.01**	**0.000**	**690**	**3.42**	**0.000**	**419**	**2.73**	**0.000**	76	Peroxisomal biogenesis factor 11 (Pex11)
7550	70	1.02	0.000	67	0.98	0.000	79	1.23	0.000	41	Peroxisome assembly protein 12 (Pex12)
9932	**968**	**2.71**	**0.000**	**1577**	**3.42**	**0.000**	**1908**	**3.70**	**0.000**	174	Peroxisomal biogenesis factor 11 (Pex11)
10503	179	1.37	0.000	198	1.52	0.000	135	0.99	0.000	83	Peroxisomal biogenesis factor 2 (Pex2)

*NC* no carbon source, *FPKM* fragments per kilobase million

### Phylogenetic study of FoxA reveals that it is well conserved in the fungal kingdom while HcsA and KatA are conserved in *Eurotiomycetes*

**Fig. 4:**
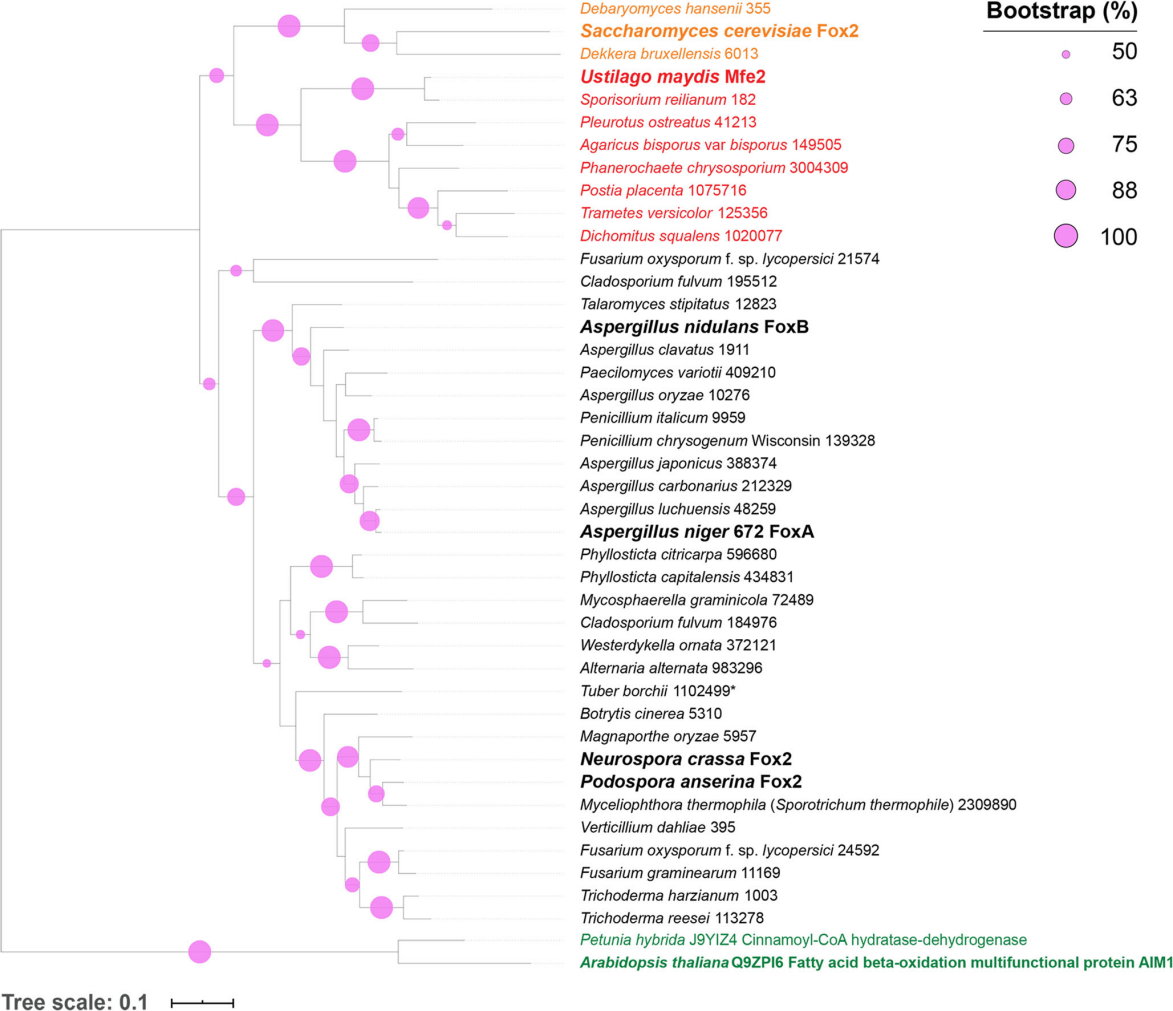
Maximum likelihood (ML; 500 bootstraps) phylogenetic tree of *A. niger* FoxA and homologs from selected fungal genomes**.** Black font represents ascomycetous fungi, red font represents basidiomycetous fungi, orange font represents *Saccharomycetes*, and green font represents plants. Enzymes in bold have been characterized. Fungal species names are followed by protein IDs from JGI (http://genome.jgi-psf.org/programs/fungi/index.jsf). Values over 50% bootstrap support are shown in pink

To verify whether HcsA, FoxA, and KatA are conserved in filamentous fungi, a phylogenetic analysis was performed. HcsA, FoxA, and KatA homologs were obtained through BLAST. FoxA homologs were studied in *A. nidulans*, *Magnaporthe oryzae*, *Neurospora crassa*, *Podospora anserina*, *Saccharomyces cerevisiae*, and *Ustilago maydis* (Fosså et al. 1995; Requena et al. 1999; Maggio-Hall and Keller 2004; Klose and Kronstad 2006; Wang et al. 2007; Boisnard et al. 2009). Most of the analyzed fungal genomes contained only one FoxA homolog (*E* ≥ −40), except for *Cladosporium fulvum* and *Fusarium oxysporum* which had an additional homolog that clustered in the same clade (Fig. 4). FoxA homologs were divided into three phylogenetic groups corresponding to fungal taxonomy.

BLASTS with HcsA as query resulted in 71 homologs, including the fatty acyl-CoA synthetases: FatA, FatB, and FatC of *A. nidulans* (Reiser et al. 2010) and Fat1 of *S. cerevisiae* (Watkins et al. 1998). All analyzed genomes of *Dothideomycetes* and *Eurotiomycetes* contained multiple homologs, while all analyzed *Sordariomycetes* and *Saccharomycotina* genomes contained one homolog. Most of the analyzed *Basidiomycetes* genomes contained no HcsA homolog except for *U. maydis* and *Sporisorium reilianum*. Phylogenetic analysis of the HcsA homologs resulted in three clusters. HcsA and FatB clustered with homologs from *Eurotiomycetes* (Supplemental Fig. S2). Localization studies of FatA and FatB revealed that both are localized in the peroxisomes, which corresponds with the prediction for the localization of HcsA.

More homologs were obtained from BLASTS with KatA as query than with HcsA. Two or more homologs were found in all analyzed genomes. Phylogenetic analysis of KatA is similar to HcsA and appeared to be well conserved in *Eurotiomycetes*, but not in other fungi, except for *Verticillium dahlia* that had a homolog clustered with KatA (Supplemental Fig. S3).

As mentioned before, *hcsA* and *katA* are neighbors on the genome. We observed that 37 out of 50 analyzed *Eurotiomycetes* have these two genes next to each other (Supplemental Table S4). In the genomes of *Monascus ruber* and *Monascus purpureus*, the homologs of *hcsA* and *katA* were separated by five genes. *Aspergillus zonatus*, *Arthroderma benhamiae*, *Coccodinium bartschii*, *Endocarpon pusillum*, *Exophiala oligosperma*, *Microsporum canis*, *Paracoccidioides brasiliensis*, *Phaeomoniella chlamydospora*, *Phaeomoniellales* sp., *Trichophyton rubrum*, and *Uncinocarpus reesii* did not have these genes as neighbors. Homologs of HcsA and KatA were also not next to each other in other fungal clades. Based on these results and the phylogenetic trees of HcsA and KatA, these enzymes are only present in *Eurotiomycetes*, suggesting that these fungi use this pathway, whereas other fungi use other pathways, or alternative genes for this pathway.

## Discussion

In fungi, it remained unclear how hydroxycinnamic acids are metabolized, since three pathways have been suggested (Baqueiro-Peña et al. 2010; Srivastava et al. 2010; Lubbers et al. 2020). Here we showed that in *A. niger*, the hydroxycinnamic acids ferulic acid, *m-*coumaric acid, *p*-coumaric acid, and caffeic acid are degraded through the peroxisomal CoA-dependent β-oxidative pathway resulting in the formation of vanillic acid, *m*-hydroxybenzoic acid, *p*-hydroxybenzoic acid, and protocatechuic acid, respectively. In addition, we showed that FoxA is not only involved in the metabolism of fatty acids but also important for the metabolism of hydroxycinnamic acids. Interestingly, this is the second observation that genes are shared between metabolism of aromatic compounds and fatty acids (Plumridge et al. 2010; Lubbers et al. 2019a).

Deletion of *hcsA*, *foxA*, or *katA* resulted in reduced growth on caffeic acid, ferulic acid, and *p*-coumaric acid. This observation supports that that these compounds are degraded through the CoA-dependent β-oxidative pathway since in other species FoxA homologs have also been shown to be part of the CoA-dependent β-oxidative conversion of fatty acids (Hiltunen et al. 1992; Maggio-Hall and Keller 2004; Boisnard et al. 2009; Gabriel et al. 2014). In addition, we observed that *m*-coumaric acid, dihydroferulic acid, dihydrocaffeic acid, and phloretic acid were also degraded through this pathway. This indicates that *m-*coumaric acid is converted to *m*-hydroxybenzoic acid. Interestingly, growth on ferulic acid was less affected by the deletion of *hcsA*, *foxA*, or *katA* compared to growth on *p-*coumaric acid and caffeic acid. Growth of Δ*hcsA* mutant on *p-*coumaric acid recovered over time, while this did not occur for Δ*foxA* and Δ*katA*. This could indicate that alternative pathways or enzymes are present for HcsA. It was suggested that *A. niger* C28B25 uses the CoA-dependent β-oxidative pathway, while the diploid strain DAR2 uses the CoA-independent oxidative pathway *A. niger* (Baqueiro-Peña et al. 2010). However, vanillin was not detected as metabolite when grown on ferulic acid (Lesage-Meessen et al. 1996; Baqueiro-Peña et al. 2010; Lubbers et al. 2020). In the closely related species *A. luchuensis*, vanillin was detected as metabolite from ferulic acid and both the CoA-independent oxidative and CoA-dependent non-oxidative pathways were suggested (Maeda et al. 2018; Taira et al. 2018). The first step of the CoA-independent oxidative pathway has been studied in *A. luchuensis* and showed that Pad was strongly induced by rice bran, but less by ferulic acid, and not by white rice or glucose (Maeda et al. 2018). In a previous study, we showed that the *A. niger* homolog of Pad (phenolic acid decarboxylase, NRRL3_8440) was induced by *p-*coumaric acid, but poorly induced by ferulic acid (Lubbers et al. 2020). It is likely that different growth conditions or higher concentrations of ferulic acid can have an effect on which pathway is activated. Therefore, we suggest that the CoA-dependent β-oxidative pathway is the main hydroxycinnamate metabolic pathway and hypothesize that at least one additional pathway, presumably the CoA-independent oxidative pathway, is present in *A. niger*.

It is known that fatty acids are converted through the CoA-dependent β-oxidative pathway and that this conversion is localized in the peroxisomes. However, no observations have been made in fungi that hydroxycinnamic acid are also converted through this pathway. In plants, hydroxycinnamic acids are converted through the CoA-dependent β-oxidative pathway located in the peroxisomes (Widhalm and Dudareva 2015). The homolog of HcsA in *A. nidulans*, named FatB (AN5877.2), has been studied (Reiser et al. 2010), and shares 73.4% amino acid sequence similarity with HcsA. Like Δ*hcsA*, the deletion of *fatB* did not result in any phenotype on fatty acids. FatB contained a C-terminal PTS1 sequence, and localization studies of FatB revealed that this protein is localized in the peroxisome (Reiser et al. 2010). The high homology of HcsA to FatB and the PTS1 sequence in its sequence strongly suggests that HcsA is localized in the peroxisome. FoxA of *A. nidulans* and *M. oryzae* and Fox2 of *P. anserina* have also been shown to be localized in the peroxisomes (Maggio-Hall and Keller 2004; Wang et al. 2007; Boisnard et al. 2009), which agrees with the localization prediction for FoxA of *A. niger*. Many peroxisomal genes were upregulated in the presence of hydroxycinnamic acids, especially two genes annotated as Pex11 proteins, which are associated with peroxisomal proliferation (Escaño et al. 2009; Wang et al. 2015). In *Penicillium chrysogenum*, three Pex11 genes (Pex11A, Pex11B, and Pex11C) were described (Opaliński et al. 2012). In *A*. *niger*, two Pex11 genes (NRRL3_9932 and NRRL3_7512, homolog of Pex11A and Pex11C, respectively) were induced by hydroxycinnamates, while NRRL3_9697 (homolog of Pex11B) was not. It has been shown that Pex11A and Pex11C of *P. chrysogenum* are localized in the peroxisomes, while Pex11B is localized in the ER (endoplasmic reticulum; Opaliński et al. 2012). Deletion of Pex11A resulted in significant loss of peroxisomes, while deletion of Pex11B and Pex11C did not. Similar results were observed in *M. oryzae* and *A. nidulans* (Hynes et al. 2008; Escaño et al. 2009; Wang et al. 2015). In *A. nidulans*, seven peroxin encoding genes (*pexA*, *pexC*, *pexE*, *pexF*, *pexG*, *pexK*, and *pexM*) were genetically studied and deletion of any of these genes resulted in abolished or severely reduced growth on fatty acids (Hynes et al. 2008). Homologs of these genes in *A. niger* (NRRL3_6923: *pexA*, NRRL3_6511: *pexC*, NRRL3_6747: *pexE*, NRRL3_7255: *pexF*, NRRL3_2754: *pexG*, NRRL3_9932: *pexK*, and NRRL3_6739: *pexM*) were also induced by caffeic acid, ferulic acid, or *p-*coumaric acid. It has been shown that in *A. nidulans*, a ketoacyl-CoA thiolase (AN1050) containing an N-terminal PTS2 is imported to peroxisomes by *pexG*, which is annotated as a PTS2 protein import receptor (Hynes et al. 2008). It is likely that the homolog of PexG in *A. niger*, NRRL3_2754, is also involved in importing KatA to the peroxisomes. Based on these results, we suggest that hydroxycinnamic acids are converted through the CoA-dependent β-oxidative in the peroxisomes.

The second and third steps of the CoA-dependent β-oxidative pathway are catalyzed by FoxA, and its homologs were also described in *Glomus mosseae*, *M. oryzae*, *N. crassa*, *P. anserina*, and *U. maydis* (Fosså et al. 1995; Requena et al. 1999; Klose and Kronstad 2006; Wang et al. 2007; Boisnard et al. 2009). Phenotypic assays were performed on fatty acids, but not on aromatic compounds. In addition, previous transcriptional studies showed that *foxA* in *A. niger* was induced by fatty acids (Maggio-Hall and Keller 2004; Klose and Kronstad 2006), while we showed that *foxA* is also induced by caffeic acid, ferulic acid, and *p-*coumaric acid. Based on the similarity between FoxA of *A. nidulans*, Fox-2 of *N. crassa*, Fox2 of *P. anserina*, and *A. niger* FoxA, we suggest that these enzymes are also involved in the peroxisomal β-oxidation of hydroxycinnamic acids. It has been shown that the deletion of *foxA* in *A. nidulans* results in abolished growth on the very long chain fatty acid erucic acid (C_22:1_), and reduced growth on the long chain fatty acid oleic acid (C_18:1_), but no phenotypes were observed on myristic acid (C_14:0_), hexanoic acid (C_6:0_), or butyric acid (C_4:0_) (Maggio-Hall and Keller 2004). Therefore, it was suggested that *foxA* is only involved in the beta oxidation of very long and long fatty acids. However, we observed that deleting *foxA* in *A. niger* results in reduced growth on oleic acid and erucic acid, but also on crotonic acid (C_4:1_). This indicates that *A. niger* FoxA is also involved in the oxidation of short-chain fatty acids. More interestingly, all tested fatty acids with phenotypes were unsaturated fatty acids. Similar results were observed in *Candida lusitaniae* when *fox2* was deleted (Gabriel et al. 2014). No growth reduction was observed on myristic acid (C_14:0_), palmitic acid (C_16:0_), and stearic acid (C_18:0_), while on oleic acid (C_18:1_) and erucic acid (C_22:1_) growth was reduced. In contrast, *C. lusitaniae* ∆*fox2* had reduced growth on lauric acid (C_12:0_). Deletion of *fox2* in *P. anserina* did not alter the growth on oleic acid (C_18:1_) (Boisnard et al. 2009). In *U. maydis*, deletion of *multifunctional enzyme 2* (*mfe2*) results in abolished growth on palmitic acid (C_16:0_) (Klose and Kronstad 2006). Therefore, alternative enzymes or pathways were suggested for the degradation of fatty acids (Boisnard et al. 2009).

In conclusion, comparative transcriptome analysis on different hydroxycinnamic acids leads to the identification of *hcsA*, *foxA*, and *katA* in *A. niger* and revealed that these genes are involved in the degradation of hydroxycinnamic acids through the peroxisomal CoA-dependent β-oxidative pathway. In addition, FoxA is not only important for the metabolism of fatty acids but it also plays a major role in the CoA-dependent β-oxidative pathway for degradation of hydroxycinnamic acids in fungi. This study contributes to a better understanding of the metabolism of aromatic compounds in fungi, including the localization of the metabolic enzymes, and unlocks new potential strategies for the production of aromatic compounds by fungi.

## Supplementary Information

ESM 1 (PDF 1350 kb)

ESM 2 (XLSX 34 kb)

## Data Availability

The datasets generated during and/or analyzed during the current study are available in the GEO repository, under accession numbers GSE134999 and GSE135001.
