# Supplementary material for: Aspergillus niger uses the peroxisomal CoA-dependent β-oxidative genes to degrade the hydroxycinnamic acids caffeic acid, ferulic acid, and p-coumaric acid
Source: Appl Microbiol Biotechnol. 2021 May 5;105(10):4199–211. doi: 10.1007/s00253-021-11311-0 (PMC8140964; doi:10.1007/s00253-021-11311-0)
Supplement: Supplementary file 1 — (PDF 1350 kb) [file 253_2021_11311_MOESM1_ESM.pdf]

Supplemental materials

Applied Microbiology and Biotechnology

Aspergillus niger uses the peroxisomal CoA-dependent β-oxidative genes to degrade the hydroxycinnamic acids caffeic acid, ferulic acid and *p*-coumaric acid

R.J.M. Lubbers, A. Dilokpimol, J. Visser and R. P. de Vries*

Fungal Physiology, Westerdijk Fungal Biodiversity Institute & Fungal Molecular Physiology, Utrecht University, Utrecht, The Netherlands

*Correspondence: R.P. de Vries (r.devries@wi.knaw.nl)

Supplemental data containing 3 figures

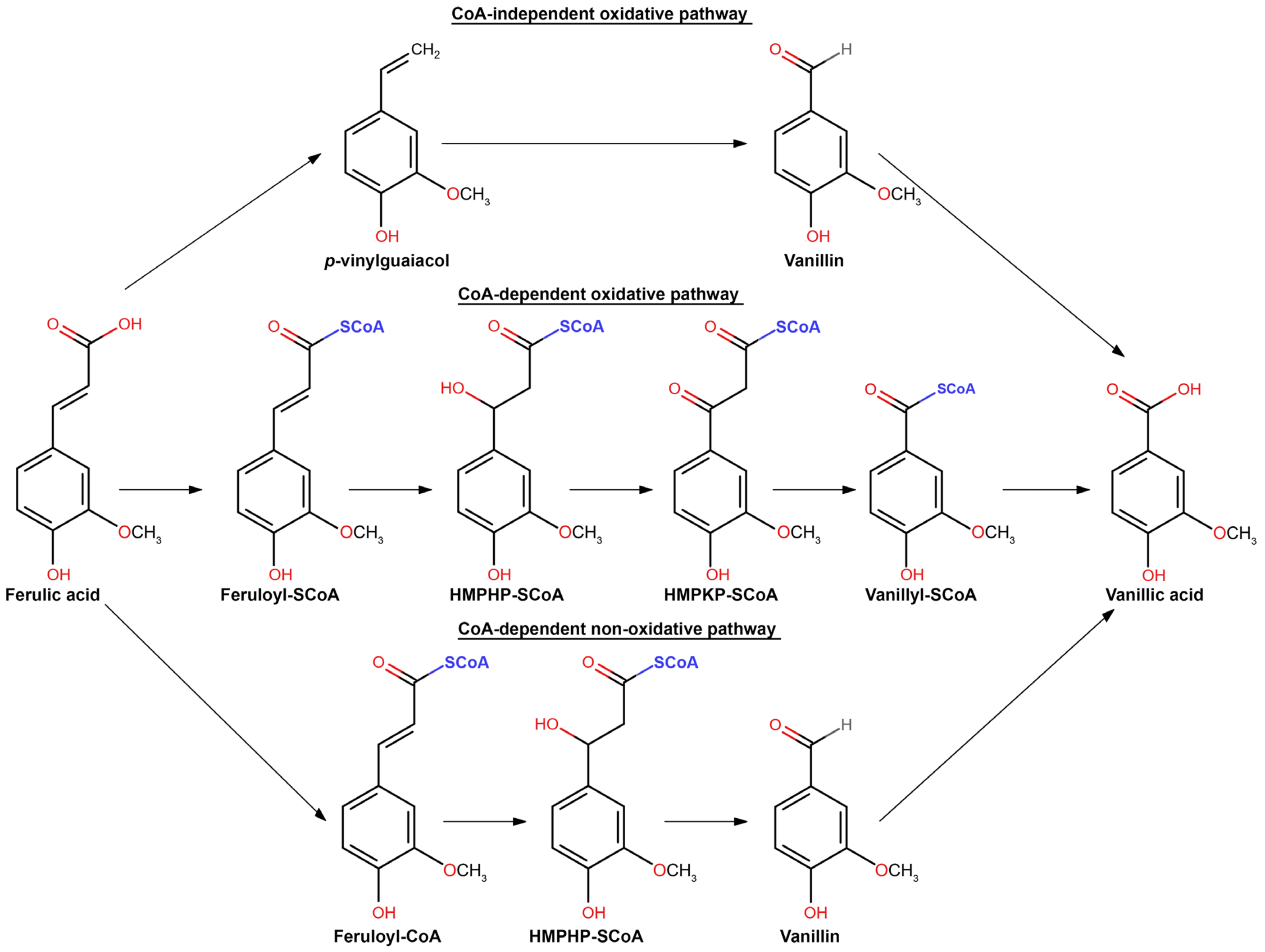

Supplemental Fig. S1. Three proposed ferulic acid metabolic pathways described in plants and microorganisms. Ferulic acid metabolic pathways were based on the studies from Priefert et al. 1999; Baqueiro-Peña et al. 2010; Srivastava et al. 2010; Otani et al. 2014; Widhalm and Dudareva 2015; Lubbers et al. 2020

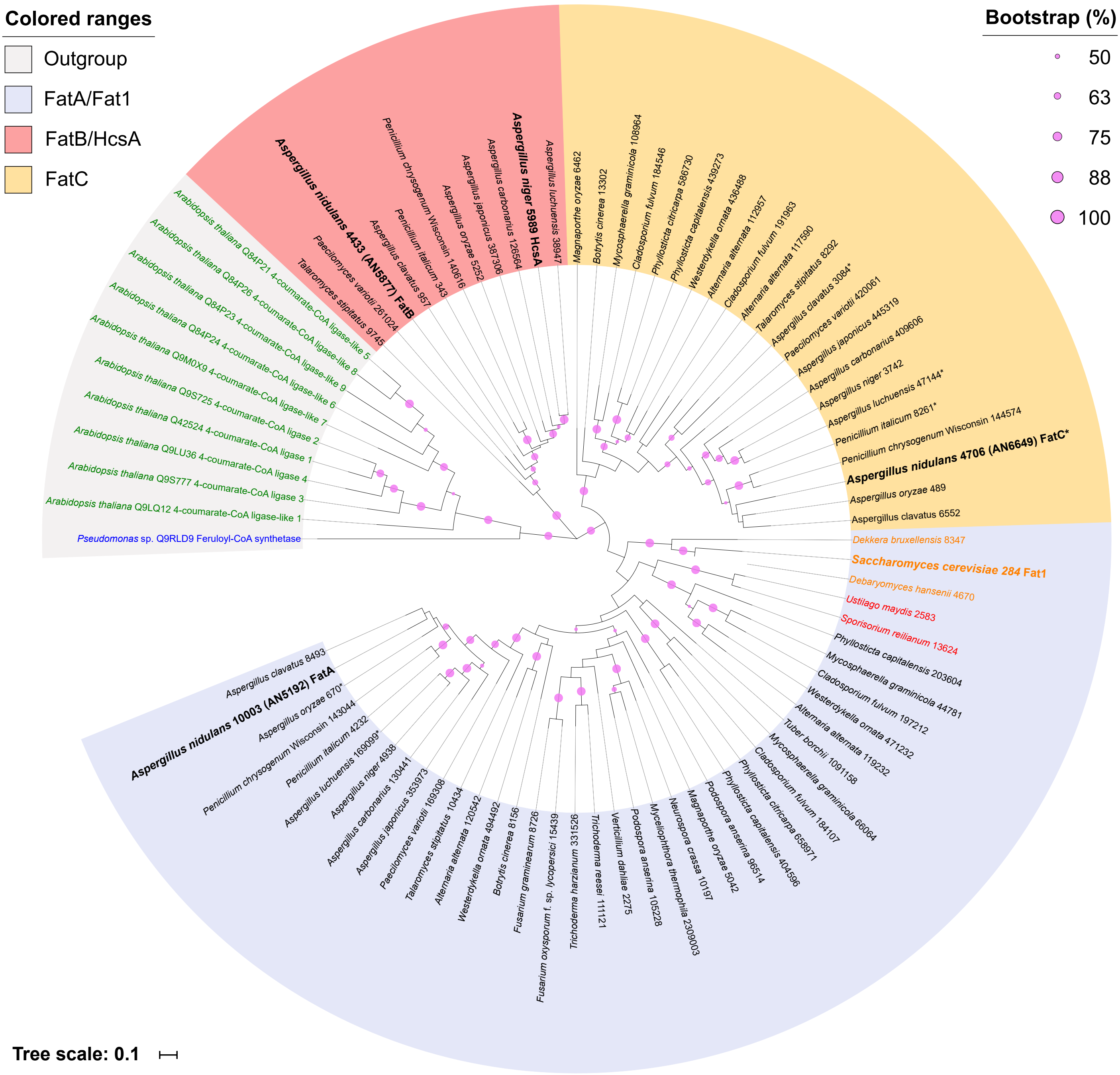

Supplemental Fig. S2. Maximum likelihood (ML; 500 bootstraps) phylogenetic tree of *A. niger* HcsA homologs from selected fungal genomes. Black font represents *Ascomycete* fungi, red font *Basidiomycete* fungi, orange font *Saccharomycetes*, blue font bacteria and green font plants. Fonts in bold are enzymes that have been characterized. Fungal species names are followed by protein IDs from JGI (http://genome.jgi-psf.org/programs/fungi/index.jsf) or from Uniprot (http://uniprot.org). Values over 50% bootstrap support are shown on the tree node with pink. The scale bar shows a distance equivalent to 0.10 amino acid substitutions per site.

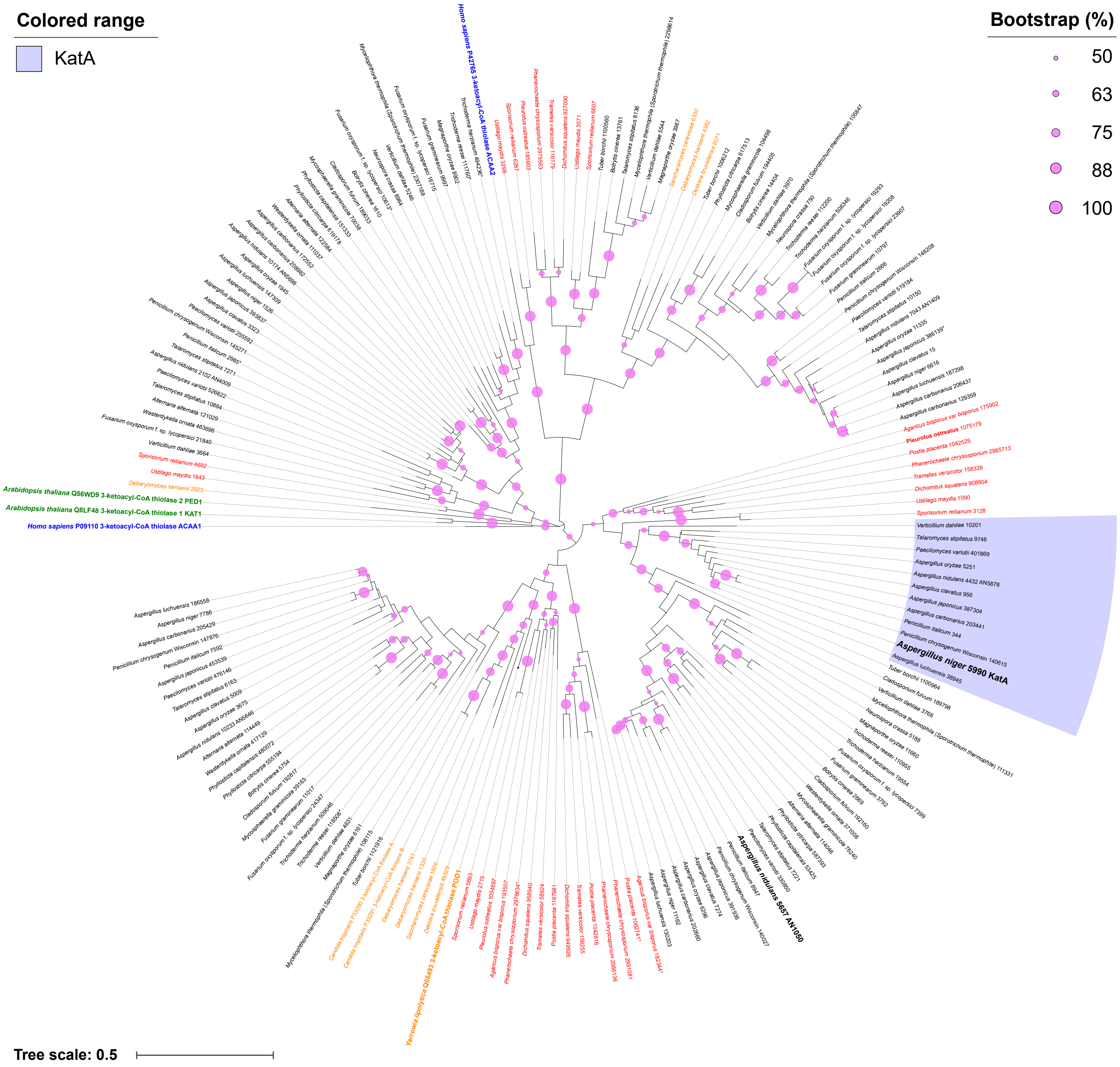

Supplemental Fig. S3. Maximum likelihood (ML; 500 bootstraps) phylogenetic tree of *A. niger* KatA homologs from selected fungal genomes. Black font represents *Ascomycete* fungi, red font *Basidiomycete* fungi, orange font *Saccharomycetes*, blue font *Homo sapiens* and green font plants. Fonts in bold are enzymes that have been characterized. Fungal species names are followed by protein IDs from JGI (http://genome.jgi-psf.org/programs/fungi/index.jsf) or from Uniprot (http://uniprot.org). Values over 50% bootstrap support are shown on the tree node with pink. The scale bar shows a distance equivalent to 0.50 amino acid substitutions per site.
